# Prognostic value of insulin resistance in patients with female reproductive system malignancies: A multicenter cohort study

**DOI:** 10.1002/iid3.1107

**Published:** 2023-12-06

**Authors:** Xiao‐Yue Liu, Qi Zhang, Xi Zhang, Yi‐Zhong Ge, Guo‐Tian Ruan, Hai‐Lun Xie, Tong Liu, Meng‐Meng Song, Li Deng, Han‐Ping Shi

**Affiliations:** ^1^ Departments of Gastrointestinal Surgery and Clinical Nutrition, Beijing Shijitan Hospital Capital Medical University Beijing China; ^2^ Beijing International Science and Technology Cooperation Base for Cancer Metabolism and Nutrition Beijing China; ^3^ Key Laboratory of Cancer FSMP for State Market Regulation Beijing China

**Keywords:** female reproductive system malignancies, inflammation, insulin resistance, metabolism, prognosis

## Abstract

**Background:**

Insulin resistance (IR) and systemic inflammation are common in patients with cancer and are associated with poor prognosis. Few studies have reported IR in female reproductive system malignancies. This study investigated the prognostic value of IR and systemic inflammation in this population.

**Methods:**

A prospective multicenter real‐world cohort study involving 571 patients diagnosed with female reproductive system malignancies was conducted. Lipid ratios (low‐density lipoprotein‐cholesterol/high‐density lipoprotein‐cholesterol [LHR], total cholesterol/HDL‐cholesterol [TCHR], triglyceride/HDL‐cholesterol [TGHR], fasting triglyceride/glucose [TyG]) were used to reflect IR. Optimal cut‐off values were determined using maximally selected rank statistics. The Kaplan–Meier and Cox regression were used to calculate the hazard ratios for overall survival.

**Results:**

Over half (55.90%) of the 571 patients with female reproductive system malignancies (mean age: 52 years) had cervical cancer. Both IR and inflammation were negatively correlated with overall survival in female reproductive system cancer patients. Multivariate survival analysis showed that patients with high LHR (hazard ratio [HR]: 1.51, 95% confidence interval [CI]: 1.01–2.25, *p* = .046), high TCHR (HR: 1.90, 95% CI:1.22–2.95, *p* = .005), high TGHR (HR: 1.66, 95% CI:1.17–2.36, *p* = .004), high TyG (HR: 1.64, 95% CI:1.13–2.40, *p* = .010), high neutrophil lymphocyte ratio (NLR, HR: 2.03, 95% CI:1.44–2.86, *p* = .004) were significantly associated with worse prognosis. By calculating the concordance index of the four IR surrogate indicators, TyG was the most valuable indicator for the prognosis of patients with malignant tumors of the female reproductive system. High TyG combined with high NLR had improved prognostic value (HR: 3.22, 95% CI: 1.97–5.26, *p* < .001).

**Conclusions:**

IR can be used as an independent predictor of prognosis in the female reproductive system malignancy population regardless of the IR substitution index. The combination of TyG and NLR could better predict the prognostic outcomes of women with breast cancer.

## Graphical abstract

This study evaluates the prognostic value of insulin resistance (IR) in female reproductive system malignancies using four surrogate markers of IR. This prognostic value is especially significant in perimenopausal women. The prognostic value of the combination of IR and inflammation status was also determined.

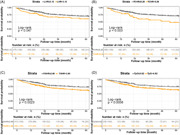

## 1 INTRODUCTION

Cervical, endometrial, and ovarian cancers are the most common cancers of the female reproductive system and cause significant cancer morbidity and mortality worldwide.^1^ As women age, hormone levels constantly change, affecting the body's metabolism. Although cancer interventions have improved significantly in recent years, the prognosis for patients with cancer remains poor. The reproductive history of women and hormonal changes play a very important role in the prognosis of patients with cancer. Therefore, identifying clinical features that reflect metabolic changes in patients and appropriate interventions may significantly improve patient outcomes.

Patients with cancer have increased insulin resistance (IR),^2^ malnutrition,^3^ and inflammatory response,^4^ leading to poorer responses to chemotherapy, increased complications, and worse prognoses. IR is a hallmark of obesity, cardiovascular disease, diabetes, and cancer.^5^ The hyperinsulinemic–euglycemic glucose clamp technique is the gold standard for the diagnosis of IR.^6^ However, due to the difficulty, high cost, and time‐consuming process of this technology, lipid ratios (low‐density lipoprotein‐cholesterol/high‐density lipoprotein‐cholesterol [LHR], total cholesterol/HDL‐cholesterol [TCHR], triglyceride/HDL‐cholesterol [TGHR], and fasting triglyceride/glucose [TyG]) have attracted increasing attention as a simple and effective alternative marker for IR.^7^, ^8^, ^9^ Recently, the TyG index has been successfully used to prove the association of nonalcoholic fatty liver disease (NAFLD) with one of the most dangerous cancers of elderly that is bladder cancer, by the mediation of the IR.^10^ In addition, the neutrophil lymphocyte ratio (NLR) is an inflammatory indicator and independent predictor of patient prognosis.^11^ IR and inflammatory state have been reported as important in the prognosis of patients with cancer. IR combined with systemic inflammation has been shown to have better prognostic value in patients with breast cancer.^12^ Clinical interventions of these markers may be an important method for the improvement of the prognosis of patients with cancer.^13^, ^14^ However, few studies regarding the prognosis of IR in patients with female reproductive system cancers have been reported.

Therefore, this study aimed to describe the prognostic value of IR in female reproductive system tumors, explore the most prognostic indicators of IR, and better evaluate the prognosis of patients by combining with inflammatory indicators.

## 2 METHODS

### 2.1 Study population

This prospective cohort study is based on the investigation on nutrition status and its clinical outcome of common cancers (INSCOC) cohort in China. The INSCOC trial was registered at http://www.chictr.org.cn under the registration number ChiCTR1800020329. The data used in this study were collected prospectively from multiple institutions in China. The design and methods of the INSCOC trial have been described previously.^15^ All patients included in the INSCOC trial were ≥18 years of age; had been diagnosed with a solid tumor; underwent surgery, chemotherapy, radiotherapy, or other anticancer therapy; and were hospitalized for >48 h. Patients with clinical evidence of active infection or immune disease and those missing certain data (such as age, height, TNM stage, fasting blood glucose, LDL cholesterol, HDL cholesterol, total cholesterol, triglycerides [TG], neutrophil count, or lymphocyte count) were excluded from the INSCOC trial. Supporting Information: Figure 1 showed the flow chart for the screening of research objects. The study followed the principles outlined in the Declaration of Helsinki and was approved by the ethics committees of the local centers. Written/oral informed consent was obtained from all patients for the use of their clinical data without disclosing personal information.

### 2.2 Data collection

Patient age, sex, primary tumor type, tumor stage, and smoking and drinking history were obtained from the electronic medical record system. Body mass index (BMI), defined as weight (kg) divided by height (m) squared, was calculated for all patients. The patients were divided into two groups: normal (≤24 kg/m^2^) and overweight/obesity (>24 kg/m^2^). The clinical staging of patients was assessed based on the TNM staging of the 8th edition of the AJCC TNM staging system.^16^ The patients’ nutritional risks (NRS2002) were assessed and recorded by trained staff at baseline. Serological indicators such as serum albumin, total cholesterol content, TG content, LDL content, HDL content, neutrophil count, lymphocyte count, and blood glucose level were obtained within 24 h after admission after an overnight fast and normalized to exclude variability due to laboratory equipment.

### 2.3 Assessment of IR and inflammatory status

The patients’ IR‐related statuses were assessed using LHR, TCHR, TGHR, and TyG, and their inflammatory response statuses were assessed using NLR based on the following formulas:

LHR: low‐density lipoprotein‐cholesterol/high‐density lipoprotein‐cholesterol
TCHR: cholesterol/high‐density lipoprotein‐cholesterol
TGHR: triglyceride/high‐density lipoprotein‐cholesterol
TyG: Ln [TG (mg/dL) × FBG (mg/dL)]/2.
NLR: neutrophil/lymphocyte
Each index was classified using maximally‐selected rank statistics to obtain the optimal cut‐off value.

### 2.4 Study endpoint

The primary endpoint of this study was all‐cause mortality. Overall survival was measured in months and was defined as the time from the date of admission to death or last follow‐up. Clinical outcome data were collected at regular follow‐up visits or via telephone.

### 2.5 Statistical analysis

Continuous variables are expressed as mean ± standard deviation or median and interquartile range (IQR). Categorical variables are presented as numbers and percentages (*n*, %). Continuous variables were compared using the independent Student *T*‐test or nonparametric test, and categorical variables were compared using the Chi‐square test or Fisher's exact test. Based on previous studies, the covariates and potential confounders were selected in advance. Hazard ratios (HRs) and 95% confidence intervals (CIs) for important prognostic factors based on overall survival were assessed using univariate and multivariate Cox regression analyses. The subgroup analysis and sensitivity analysis were performed. Restricted cubic splines were used to explore the association between IR‐related indices and survival in patients with malignancies of the female reproductive system. Kaplan–Meier curves and log‐rank tests were used to present time–patient survival trends and compare survival between groups. The Harrell C index was calculated to evaluate and compare the predictive ability of IR indexes on patient survival. We also used the receiver operating characteristic curve to compare the prognostic value of TGHR and NLR combined with TGHR and NLR alone for female reproductive system malignancies. A two‐sided *p* value of <.05 was considered statistically significant. All statistical analyses were performed using R software, version 4.1.1 (https://www.r-project.org/).

## 3 RESULTS

### 3.1 Patient characteristics

A total of 571 patients diagnosed with female reproductive malignancies were included in this study. The mean patient age was 52.0 ± 14.0 years (Table 1). The most common cancer was cervical cancer (319, 55.90%), and 361 (63.20%) of patients underwent systemic chemotherapy and 112 (19.60%) of the patients were at nutritional risk. Compared with the deleted 1041 patients, the patients included in the study had slightly higher hypertension, chemotherapy, albumin, fasting blood glucose, BMI, and less surgery and risk of nutrition.

**Table 1:** Baseline characteristics of the study population.

Characteristics	*n* = 1041^a^	*n* = 571^b^	*p* Value
Age	52.00 [46.00, 59.00]	52.00 [46.00, 60.00]	.102
Diabetes, yes	63 (6.10)	34 (6.00)	1.000
Hypertension, yes	130 (12.50)	94 (16.50)	.033
Family history of cancer, yes	141 (13.50)	94 (16.50)	.130
Smoking, yes	55 (5.30)	33 (5.80)	.761
Drinking, yes	15 (1.40)	17 (3.00)	.054
Tumor stage			.090
I	211 (25.80)	129 (22.60)	
II	200 (24.40)	155 (27.10)	
III	209 (25.60)	125 (21.90)	
IV	198 (24.20)	162 (28.40)	
Tumor type			.227
Cervical cancer	542 (52.10)	319 (55.90)	
Ovarian cancer	338 (32.50)	179 (31.30)	
Endometrial cancer	161 (15.50)	73 (12.80)	
Surgery, Yes	321 (30.80)	127 (22.20)	<.001
Chemotherapy, yes	565 (54.30)	361 (63.20)	.001
Radiotherapy, yes	130 (12.50)	72 (12.60)	1.000
Albumin	40.00 [36.30, 43.40]	40.65 [37.30, 43.70]	.022
CRP	6.48 [1.70, 31.10]	4.98 [2.32, 18.75]	.282
FBG	5.08 [4.62, 5.69]	5.16 [4.80, 5.76]	.002
Hb	113.00 [100.00, 125.00]	113.50 [103.00, 126.00]	.281
Neutrophils	3.40 [2.31, 5.39]	3.40 [2.37, 4.67]	.316
BMI	22.60 [20.30, 24.83]	23.20 [21.10, 25.40]	.001
TSF	20.00 [14.00, 26.00]	19.00 [13.00, 25.00]	.154
NRS2002			<.001
<3	748 (71.90)	459 (80.40)	
≥3	293 (28.10)	112 (19.60)	

*Note*: Values are mean (SD) or *n* (%).

Abbreviations: BMI, body mass index; CRP, C‐reactive protein; FBG, fasting blood‐glucose; Hb, hemoglobin; NRS2002, nutrition risk screening; TSF, Triceps fold thickness.

^a^ People deleted due to missing data.

^b^ The population included in this study.

### 3.2 IR, inflammatory indicators, and prognosis

**Figure 1:**
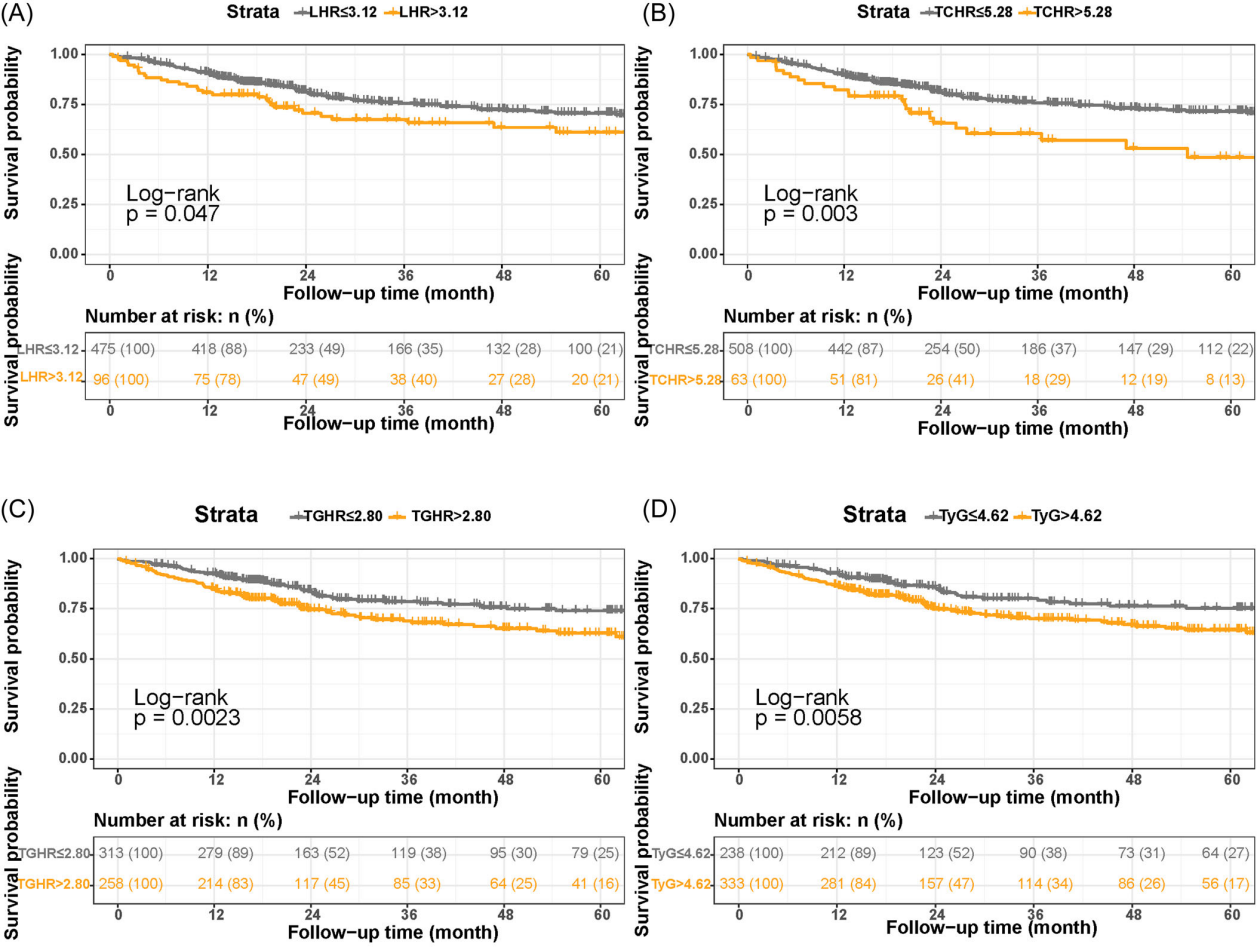
Kaplan–meier curves of all‐cause mortality by insulin resistance classification in women with cancer of the reproductive system. (A) LHR. (B) TCHR. (C) TGHR. (D) TyG. LHR, low‐density lipoprotein‐cholesterol/high‐density lipoprotein‐cholesterol; TCHR, total cholesterol to HDL‐cholesterol; TGHR, triglyceride to HDL‐cholesterol; TyG, fasting triglyceride‐glucose.

First, we analyzed the correlation between IR and prognosis using a restricted spline curve, and the results showed that IR was negatively correlated with prognosis in women with reproductive system tumors (Supporting Information: Figure 2). Since there is no uniform cut point value for lipid ratio in the current study, we classified each index by using maximally selected rank statistics to obtain the optimal cut‐off point value (Supporting Information: Figure 3). Kaplan–Meier curves were used to explore the relationship between IR status and overall survival of patients (Figure 1). Higher IR levels were significantly associated with poorer prognosis of patients.

Univariate predictors of mortality in this study population are shown in Supporting Information: Table 1. Regardless of the IR index used, we found that higher IR was associated with higher mortality. This relationship was significant when the index was used as a classification variable, and was statistically significant in uncorrected and different multifactor corrected models (Table 2). Multivariate survival analysis showed that patients with high LHR (HR: 1.51, 95% confidence interval [CI]: 1.01–2.25, *p* = .046), high TCHR (HR: 1.90, 95% CI: 1.22–2.95, *p* = .005), high TGHR (HR: 1.66, 95% CI:1.17–2.36, *p* = .004), high TyG (HR: 1.64, 95% CI:1.13–2.40, *p* = .010) were associated with worse prognosis. NLR was also negatively associated with overall patient survival. Patients with female reproductive system tumors with high NLR had a 2.03‐fold elevated risk of all‐cause mortality compared to patients with low inflammation (HR: 2.03, 95% CI:1.44–2.86, *p* = .004, Table 2).

**Table 2:** Cox proportional analysis of insulin resistance to predict all‐cause mortality from patients with female reproductive malignancies.

	Crude HR (95% CI)	*p* Value	Adjusted HR (95% CI)^a^	*p* value	Adjusted HR (95% CI)^b^	*p* Value	Adjusted HR (95% CI)^c^	*p* Value
LHR, as continues	1.01 (0.85, 1.20)	.895	0.94 (0.79, 1.12)	.499	0.93 (0.78, 1.1)	.385	1.01 (0.85, 1.20)	.918
Category								
Low LHR	Ref							
High LHR	1.50 (1.00, 2.23)	.049	1.49 (1, 2.22)	.052	1.46 (0.98, 2.2)	.066	1.51 (1.01, 2.25)	.046
TCHR, as continues	1.2 (1.02, 1.41)	.032	1.08 (0.91, 1.29)	.368	1.04 (0.88, 1.24)	.641	1.19 (1.01, 1.4)	.043
Category								
Low TCHR	Ref							
High TCHR	1.93 (1.24, 2.99)	.004	1.47 (0.94, 2.29)	.092	1.36 (0.87, 2.13)	.175	1.9 (1.22, 2.95)	.005
TGHR, as continues	1.11 (0.95, 1.3)	.183	1.01 (0.87, 1.19)	.866	1 (0.85, 1.17)	.965	1.09 (0.93, 1.29)	.281
Category								
Low TGHR	Ref							
High TGHR	1.68 (1.20, 2.36)	.003	1.5 (1.07, 2.11)	.019	1.43 (1.02, 2.02)	.039	1.66 (1.17, 2.36)	.004
TyG, as continues	1.20 (1.03, 1.4)	.022	1.12 (0.96, 1.3)	.155	1.11 (0.95, 1.3)	.192	1.19 (1.01, 1.41)	.043
Category								
Low TyG	Ref							
High TyG	1.66 (1.15, 2.38)	.006	1.48 (1.03, 2.14)	.035	1.49 (1.02, 2.17)	.039	1.64 (1.13, 2.4)	.010
NLR, as continues	1.2 (1.04, 1.38)	.012	1.19 (1.03, 1.38)	.017	1.11 (0.95, 1.3)	.198	1.21 (1.05, 1.4)	.007
Category								
Low NLR	Ref							
High NLR	1.96 (1.4, 2.76)	<.001	1.69 (1.2, 2.38)	.003	1.45 (1.01, 2.08)	.041	2.03 (1.44, 2.86)	<.001

Abbreviations: LHR, low‐density lipoprotein‐cholesterol/high‐density lipoprotein‐cholesterol; NLR, neutrophil lymphocyte ratio; TCHR, total cholesterol to HDL‐cholesterol; TGHR, triglyceride to HDL‐cholesterol; TyG, fasting triglyceride‐glucose.

^a^ Adjusted by age, tumor stage.

^b^ Adjusted by age, tumor stage, surgery, chemotherapy, radiotherapy, albumin.

^c^ Adjusted by diabetes, hypertension, BMI.

Supporting Information: Table 2 summarizes the comparison of surrogate measures of IR. The clinical significance of the four IR indexes was compared by c‐index analysis. TyG had the highest prognostic value (C‐index 0.55, 95% CI: 0.50–0.60). We also analyzed the prognostic value of TyG in different subgroups and found high TyG in patients aged 45–55 or <65 years, without metabolic disease (diabetes, hypertension, obesity), smoking, and cervical cancer, and TNM stage III patients with malignant tumors of the female reproductive system had worse survival (Table 3).

**Table 3:** Subgroup analysis.

Characteristics	TyG ≤ 4.62 (*n* = 238)	TyG > 4.62 (*n* = 333)	HR (95% CI)	*p* Value	*p* for interaction
Age					.908
<45	60 (25.21)	49 (14.71)	0.92 (0.36, 2.36)	.864	
45–55	100 (42.02)	147 (44.14)	2.36 (1.21, 4.61)	.012	
>55	78 (32.77)	137 (41.14)	1.29 (0.74, 2.25)	.362	
Age					.533
<65	207 (86.97)	284 (85.29)	1.65 (1.08, 2.52)	.020	
≥65	31 (13.03)	49 (14.71)	0.95 (0.41, 2.24)	.915	
Diabetes					.526
No	233 (97.90)	304 (91.29)	1.45 (0.98, 2.13)	.060	
Yes	5 (2.10)	29 (8.71)	0.08 (0, 3.03)	.174	
Hypertension					.271
No	212 (89.08)	265 (79.58)	1.6 (1.06, 2.39)	.024	
Yes	26 (10.92)	68 (20.42)	0.97 (0.28, 3.3)	.956	
BMI					.085
≤24	163 (68.49)	181 (54.35)	1.81 (1.1, 2.98)	.019	
>24	75 (31.51)	152 (45.65)	1.08 (0.59, 1.97)	.813	
Smoking					.705
No	229 (96.22)	309 (92.79)	1.42 (0.97, 2.09)	.075	
Yes	9 (3.78)	24 (7.21)	53.82 (2.12, 1367.87)	.016	
Tumor type					.050
Cervical cancer	144 (60.50)	175 (52.55)	2.19 (1.21, 3.96)	.010	
Ovarian cancer	70 (29.41)	109 (32.73)	1.18 (0.7, 2)	.533	
Endometrial cancer	24 (10.08)	49 (14.71)	0.74 (0.15, 3.67)	.711	
Tumor stage					.630
I	54 (22.69)	75 (22.52)	1.61 (0.48, 5.42)	.441	
II	70 (29.41)	85 (25.53)	1.65 (0.42, 6.53)	.472	
III	59 (24.79)	66 (19.82)	2.09 (1.01, 4.32)	.046	
IV	55 (23.11)	107 (32.13)	1.27 (0.76, 2.12)	.361	

*Note*: Adjusted by age, tumor stage, surgery, chemotherapy, radiotherapy, albumin.

Abbreviations: BMI, body mass index; HR, hazard ratio; TyG, fasting triglyceride‐glucose

### 3.3 Prognostic value of IR combined with inflammation

In 571 eligible patients, 16.6% had higher TyG and NLR. The Kaplan–Meier curve showed that patients with high TyG and NLR had the worst survival, while those with low TyG and NLR had the longest survival (log‐rank *p* < .0001, Supporting Information: Figure 4). Neither in unadjusted COX survival analysis (HR: 3.29; 95% CI: 2.02, 5.35; *p* < .001) nor adjusted for age, tumor stage, surgery, chemotherapy, radiotherapy, albumin (HR: 2.09; 95% CI: 1.26, 3.45; *p* = .004, Table 4), high IR combined with high inflammatory state were both identified as adverse prognostic factors affecting the survival of patients with malignant tumors of the female reproductive system. Subsequently, we also adjusted for diabetes, hypertension, and BMI separately to reduce the confounding effect of metabolic factors, and showed that the above results were still statistically significant (HR: 3.22; 95% CI: 1.97, 5.26; *p* < .001).

**Table 4:** Cox proportional analysis of insulin resistance combined with inflammatory response markers predicts all‐cause mortality.

	Crude HR (95% CI)	*p* Value	Adjusted HR (95% CI)^a^	*p* Value	Adjusted HR (95% CI)^b^	*p* Value	Adjusted HR (95% CI)^c^	*p* Value
TyGNLR (Total patients)								
Group N	Ref.		Ref.		Ref.		Ref.	
Group infla	1.43 (0.77, 2.66)	.256	1.28 (0.69, 2.37)	.441	1.04 (0.55, 1.95)	.905	1.44 (0.78, 2.69)	.245
Group IR	1.39 (0.87, 2.22)	.165	1.27 (0.79, 2.02)	.322	1.21 (0.75, 1.95)	.426	1.35 (0.84, 2.17)	.222
Group A	3.29 (2.02, 5.35)	<.001	2.49 (1.52, 4.07)	<.001	2.09 (1.26, 3.45)	.004	3.22 (1.97, 5.26)	<.001
TyGNLR (sensitivity analysis)								
Group N	Ref.		Ref.		Ref.		Ref.	
Group infla	1.49 (0.75, 2.98)	.255	1.35 (0.68, 2.70)	.395	1.19 (0.59, 2.42)	.624	1.54 (0.77, 3.08)	.225
Group IR	1.49 (0.88, 2.51)	.135	1.41 (0.84, 2.39)	.197	1.29 (0.75, 2.2)	.352	1.45 (0.85, 2.48)	.175
Group A	2.90 (1.64, 5.14)	<.001	2.28 (1.28, 4.06)	.005	2 (1.11, 3.61)	.022	2.87 (1.61, 5.12)	<.001
TyGNLR (cervical cancer)								
Group N	Ref.		Ref.		Ref.		Ref.	
Group infla	2.29 (0.88, 5.95)	.088	1.96 (0.75, 5.09)	.168	1.52 (0.57, 4.03)	.404	2.25 (0.86, 5.84)	.097
Group IR	1.69 (0.75, 3.8)	.204	1.77 (0.78, 4.03)	.171	1.79 (0.78, 4.13)	.172	1.71 (0.75, 3.92)	.203
Group A	6.29 (2.91, 13.56)	<.001	4.63 (2.11, 10.14)	<.001	3.75 (1.64, 8.55)	.002	6.28 (2.9, 13.62)	<.001
TyGNLR(Ovarian Cancer)								
Group N	Ref.		Ref.		Ref.		Ref.	
Group infla	0.68 (0.26, 1.73)	.416	0.70 (0.27, 1.8)	.463	0.66 (0.25, 1.72)	.399	0.66 (0.26, 1.69)	.387
Group IR	1.11 (0.61, 2.03)	.735	1.09 (0.60, 1.99)	.779	0.99 (0.54, 1.84)	.982	0.92 (0.48, 1.74)	.788
Group A	1.71 (0.82, 3.57)	.150	1.55 (0.74, 3.23)	.245	1.38 (0.65, 2.92)	.404	1.51 (0.71, 3.21)	.283
TyGNLR (endometrial cancer)								
Group N	Ref.		Ref.		Ref.		Ref.	
Group infla	4.98 (0.69, 35.73)	.111	6.39 (0.77, 52.72)	.085	0.55 (0.02, 15.66)	.726	7.12 (0.88, 57.73)	.066
Group IR	0.75 (0.13, 4.53)	.758	0.59 (0.09, 3.71)	.576	0.44 (0.06, 3.06)	.408	0.62 (0.09, 4.3)	.631
Group A	3.64 (0.7, 18.84)	.123	2.13 (0.39, 11.65)	.385	1.82 (0.28, 11.74)	.529	4.69 (0.87, 25.32)	.072

Abbreviations: HR, hazard ratio; NLR, neutrophil lymphocyte ratio; TyG, fasting triglyceride‐glucose.

^a^ Adjusted for age, tumor stage.

^b^ Adjusted for age, tumor stage, surgery, chemotherapy, radiotherapy, albumin.

^c^ Adjusted for diabetes, hypertension, BMI.

When patients who had died within 6 months were excluded from the analysis, high IR combined with high inflammatory status predicted a low overall survival. High IR combined with high inflammatory status was significantly associated with poor overall survival in patients with cervical cancer. The same trend was noted for patients with ovarian and endometrial cancers after multivariable adjustment, though the trend was not significant in these groups.

The prognostic ability of IR combined with inflammatory response indicators was stronger than that of the separate prognostic abilities of TyG and NLR (Supporting Information: Figure 5), and the difference was statistically significant (AUC TyG+NLR: 0.646; TyG: 0.576; NLR: 0.622). A difference of 0.025 from the reference was considered a better discrimination.^17^

## 4 DISCUSSION

In this multicenter, retrospective study, 571 women with malignant tumors of the reproductive system were included. Lipid ratios have been reported as predictors of IR in patients with different glucose tolerance levels.^18^ In all, 11.2%–45.5% patients were found to have IR according to the five lipid ratios. The prognosis of patients with cancer depends not only on the tumor itself but also on the metabolic alterations and inflammatory responses of the body caused by the tumor status. The results of the present study confirm that IR and systemic inflammation are strongly associated with overall survival in patients with malignant tumors of the female reproductive system. In addition, IR combined with inflammatory markers better predicts the prognoses of patients.

IR is a multifactorial disorder characterized by the decreased ability of insulin to regulate glucose homeostasis.^19^ The prognostic value of IR status in different tumors has been reported. The presence of IR is associated with the progression of lung cancer.^20^ IR is also associated with a poor prognosis in African American and Caucasian women with breast cancer^21^ and is positively associated with postoperative recurrence.^22^ IR is significantly associated with later tumor stages in men with prostate cancer.^23^ However, few studies regarding the relationship between IR and the prognoses of patients with malignant tumors in the female reproductive system have been reported. The results of this study indicate that IR can also be used as an independent prognostic factor in this patient population.

Systemic inflammatory biomarkers are considered to be hallmarks of cancer and cost‐effective prognostic factors,^24^ and NLR is a recognized marker of systemic inflammation.^25^ Neutrophils play an important role in acute inflammatory responses, and lymphocytes are key cells in the host cytotoxic immune response and play a crucial role in the cell‐mediated antitumor microenvironment.^26^ NLR has been reported as an independent prognostic factor in patients with cancer.^27^ The systemic inflammatory response increases in patients with cancer, leading to increase secretion of inflammatory mediators from adipose tissue, such as tumor necrosis factor α, interleukin (IL)‐1β, and IL‐6.^28^ These cytokines impair the phosphorylation of insulin receptors and insulin receptor substrate 1 by inducing the expression of SOCS‐3, a potential inhibitor of insulin signaling, leading to decreased insulin sensitivity of adipocytes and promotion of IR occur.^29^, ^30^

Insulin has a variety of metabolic functions. In addition to the most basic hypoglycemic effect,^31^ it can also be used as a growth factor affecting cell proliferation.^32^ Insulin has mitotic functions in normal breast tissue and in breast cancer cells.^33^ The occurrence of IR in patients with cancer is associated with genetic and environmental factors, as well as systemic inflammation.^34^ As the gold standard for IR diagnosis is difficult to achieve clinically, lipid ratios that can be used as surrogate indicators have received increasing attention from researchers. In this study, TyG was determined as the best indicator to reflect the IR status of patients with malignant tumors of the female reproductive system. This is consistent with previous research by Tarantino et al.^10^ TyG combined with NLR to reflect the body's insulin metabolism‐related levels as well as the immune inflammation status. This combination was identified as an independent prognostic factor for patients with female reproductive system malignancies regardless in the univariate survival analysis and after adjustment for confounders.

In the subgroup analysis, we found an interesting result: the prognosis of perimenopausal female reproductive system malignancies with IR was significantly worse than that of reproductive and postmenopausal women. The findings of this result appear to be related to the specific metabolic changes in women during perimenopause. Studies had showed that the transport protein, serum sex hormone binding globulin (SHBG), is a strong independent marker of IR.^35^ Importantly, the relationship between SHBG and IR in postmenopausal women is independent of both endogenous estrogens and androgens.^36^ There were also animal experiments which showed that mice were subjected to simulated menopause operations—ovariectomized. Ovariectomized mice showed reduced energy expenditure but no subsequent changes in energy intake, leading to the development of fat cell hypertrophy, adipose tissue inflammation, and IR.^37^

This study is the first to explore the relationship between IR, inflammation, and prognosis in a representative group of Chinese female patients with reproductive system malignancies. However, this study has several limitations. First, this study was based on the Chinese population and excluded most people due to lack of data. As the lifestyles of patients differ globally, these results should be verified in a more diverse patient population. Second, although we have adjusted for known confounders wherever possible, potential confounders may still exist and visceral fat was not adjusted due to the lack of such data in this database. Previous studies have shown that centrally accumulation of body fat is associated with IR.^38^ Third, data regarding some female‐specific predictors, such as sex hormones, were not collected at the beginning of the study, and only patient‐related indicators collected at admission were assessed, without dynamic monitoring of these indicators.

## 5 CONCLUSIONS

In conclusion, IR can be used as an independent prognostic factor of female reproductive system malignancy regardless of the alternative index of IR, and the prognostic value is related to whether the patient is in perimenopause. Therefore, the assessment and treatment of IR may be an important component of determining the prognosis of patients with cancer.

## AUTHOR CONTRIBUTIONS

Han‐Ping Shi and Li Deng contributed to the design of the research; Qi Zhang, Xi Zhang, and Guo‐Tian Ruan contributed to the interpretation of the data; Xiao‐Yue Liu, Hai‐Lun Xie, Yi‐Zhong Ge, and Meng‐Meng Song contributed to data acquisition and analysis. Xiao‐Yue Liu and Tong Liu drafted the manuscript. All authors critically revised the manuscript, agreed to be fully accountable for ensuring the integrity and accuracy of the work, and read and approved the final manuscript.

## CONFLICT OF INTEREST STATEMENT

The authors declare no conflict of interest.

## ETHICS STATEMENT

The study followed the principles outlined in the Declaration of Helsinki and was approved by the Medical Ethics Committee of Beijing Shijitan Hospital, Capital Medical University. Written informed consent to use clinical data without disclosing personal information was obtained from each patient.

## Supporting information

Supporting information.

## Data Availability

All data needed to evaluate the conclusions of the study are presented in this paper and/or the Supplementary Materials. Additional data related to this study are requested from the authors. If someone wants to request the data from this study should contact lxy304305765@163.com.
