# Supplementary material for: Prognostic value of insulin resistance in patients with female reproductive system malignancies: A multicenter cohort study
Source: Immun Inflamm Dis. 2023 Dec 6;11(12):e1107. doi: 10.1002/iid3.1107 (PMC10698827; doi:10.1002/iid3.1107)

Supplementary table 1. Univariable Cox regression analyses of factors predicting all-cause mortality.

| **Characteristics** | HR(95%CI) | *P*-value |
| --- | --- | --- |
| **Age** | 1.03 (1.01,1.05) | 0.001 |
| **Diabetes** |  |  |
| **No** | Ref. |  |
| **Yes** | 1.31 (0.67,2.58) | 0.434 |
| **Hypertension** |  |  |
| **No** | Ref. |  |
| **Yes** | 1.21 (0.77,1.89) | 0.411 |
| **Family history of cancer** | |  |
| **No** | Ref. |  |
| **Yes** | 0.72 (0.44,1.16) | 0.178 |
| **Smoking** |  |  |
| **No** | Ref. |  |
| **Yes** | 1.19 (0.62,2.26) | 0.602 |
| **Drinking** |  |  |
| **No** | Ref. |  |
| **Yes** | 0.19 (0.03,1.32) | 0.093 |
| **Tumor stage** |  |  |
| **I** | Ref. |  |
| **II** | 0.68 (0.30,1.51) | 0.343 |
| **III** | 3.31 (1.76,6.22) | <0.001 |
| **IV** | 6.73 (3.71,12.2) | <0.001 |
| **Surgery** |  |  |
| **No** | Ref. |  |
| **Yes** | 0.27 (0.14,0.51) | <0.001 |
| **Chemotherapy** |  |  |
| **No** | Ref. |  |
| **Yes** | 1.91 (1.29,2.82) | 0.001 |
| **Radiotherapy** |  |  |
| **No** | Ref. |  |
| **Yes** | 0.53 (0.3,0.95) | 0.032 |
| **Albumin** | 0.95 (0.92,0.98) | <0.001 |
| **LHR, as continuous** | 1.1 (0.91,1.33) | 0.331 |
| **Category** |  |  |
| **Low LHR** | Ref. |  |
| **High LHR** | 1.50(1.00,2.23) | 0.049 |
| **TCHR, as continuous** | 1.16 (1.01,1.33) | 0.030 |
| **Category** |  |  |
| **Low TCHR** | Ref. |  |
| **High TCHR** | 1.93 (1.24,2.99) | 0.004 |
| **TGHR, as continuous** | 1.03 (0.98,1.09) | 0.195 |
| **Category** |  |  |
| **Low TGHR** | Ref. |  |
| **High TGHR** | 1.68 (1.2,2.36) | 0.003 |
| **TyG, as continuous** | 1.69 (0.96,2.97) | 0.070 |
| **Category** |  |  |
| **Low TyG** | Ref. |  |
| **High TyG** | 1.66 (1.15,2.38) | 0.006 |
| **NLR, as continuous** | 1.03 (1,1.07) | 0.058 |
| **Category** |  |  |
| **Low NLR** | Ref. |  |
| **High NLR** | 1.96 (1.4,2.76) | <0.001 |
| **BMI, as continuous** | 1.02 (0.96,1.08) | 0.562 |
| **Category** |  |  |
| **≤24** | Ref. |  |
| **>24** | 1.18 (0.84,1.65) | 0.342 |

Supplementary table 2. C-index of indexs.

|  | c-index | lower .95 | upper .95 |
| --- | --- | --- | --- |
| TyG | 0.5543745 | 0.5040862 | 0.6046629 |
| TGHR | 0.5508226 | 0.4998654 | 0.6017797 |
| TCHR | 0.5366325 | 0.4831292 | 0.5901358 |
| LHR | 0.5025282 | 0.4472686 | 0.5577878 |

Supplementary Figure 1. Flow chart.


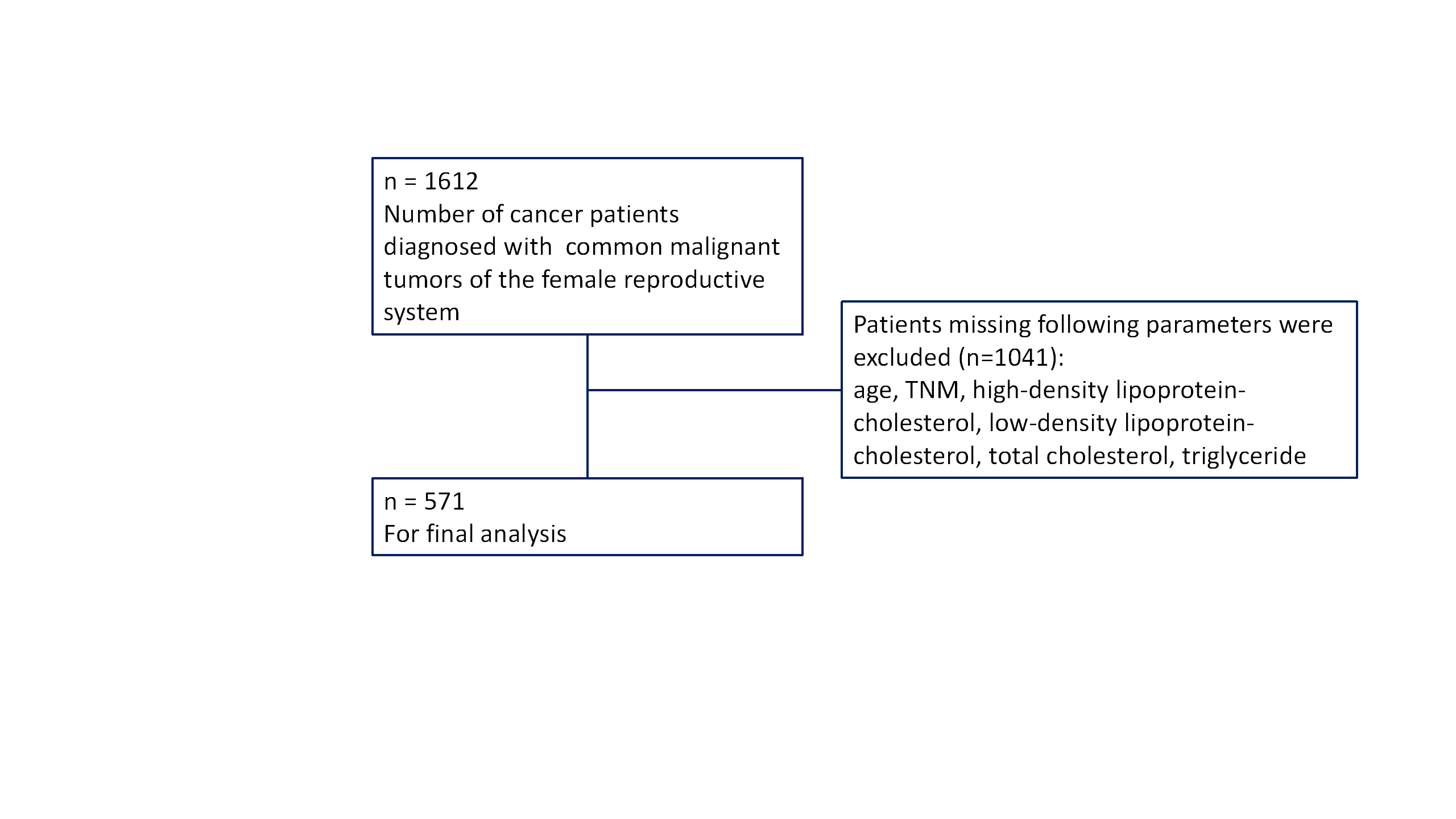


Supplementary Figure 2. Correlation between insulin resistance and prognosis of female reproductive malignancies


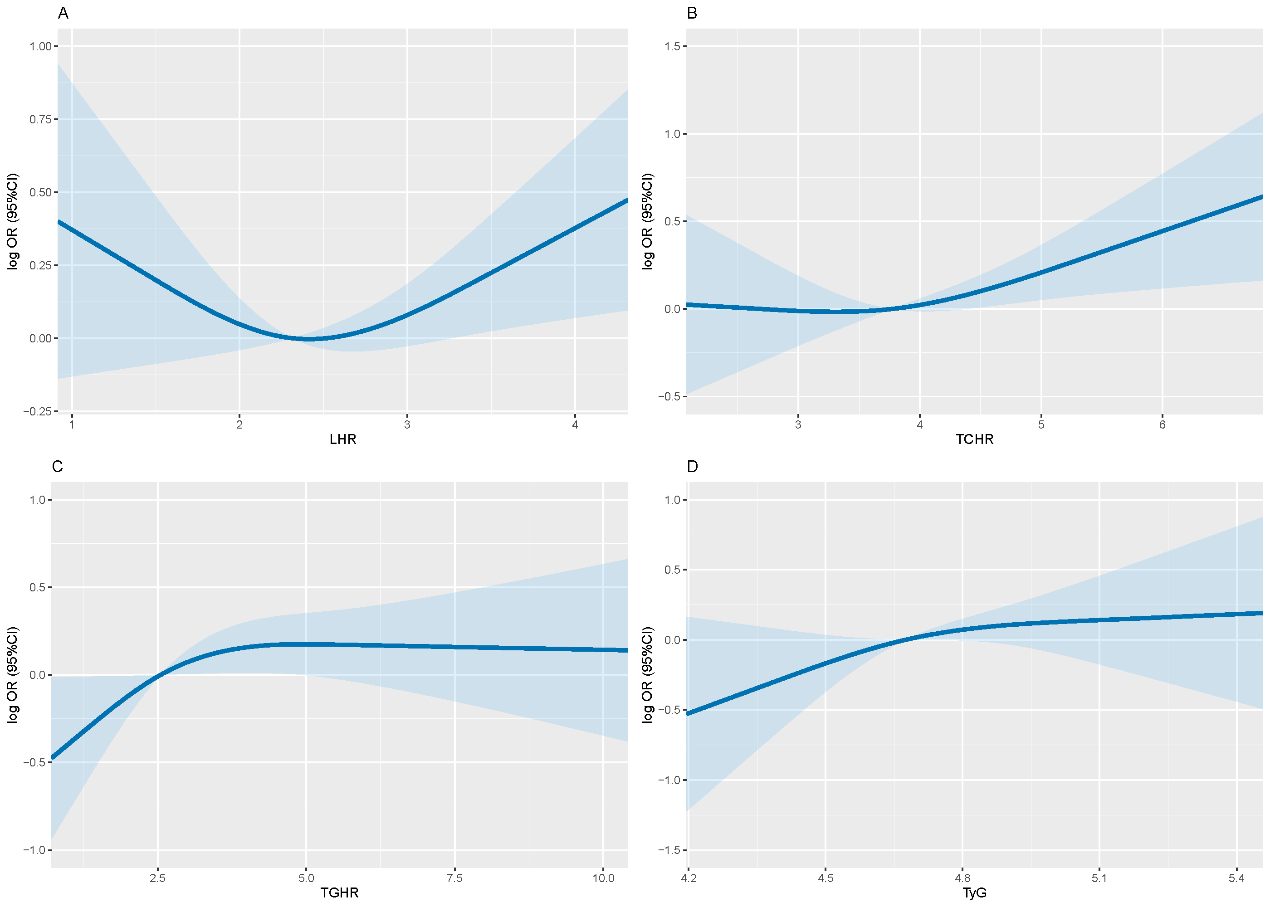


Supplementary Figure 3. Cut-off value of each indicator.


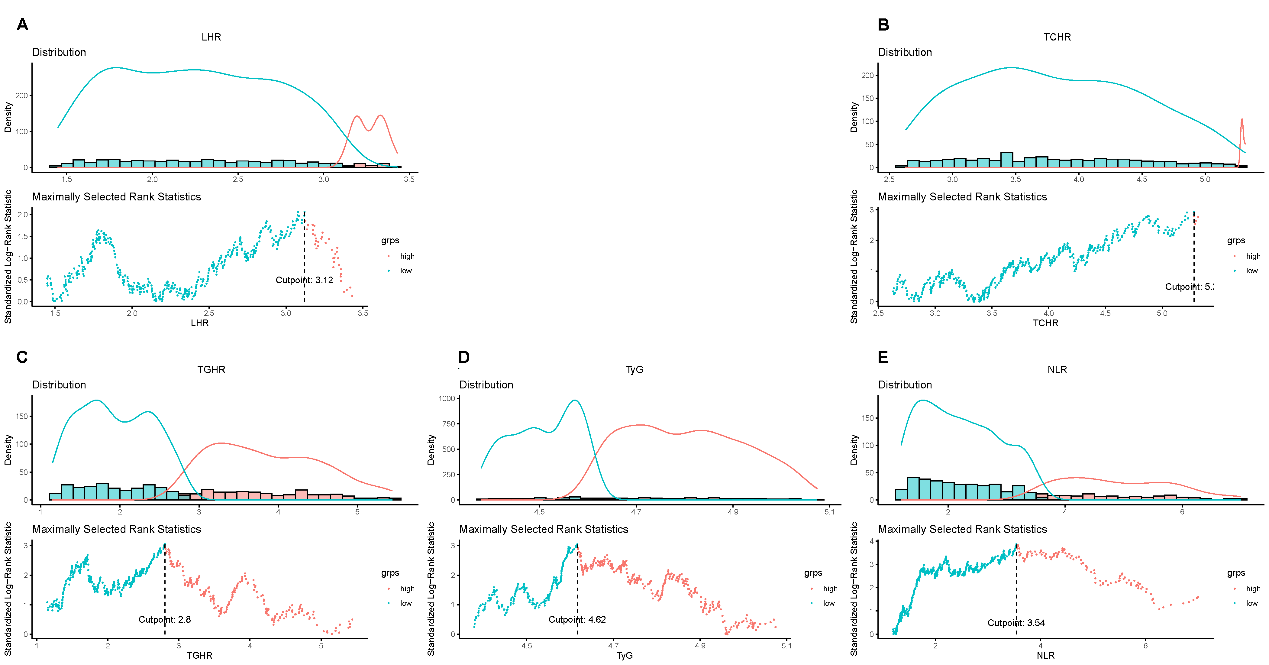


Supplementary Figure 4. Kaplan-meier curves of all-cause mortality by TyG and NLR classification in women with cancer of the reproductive system.


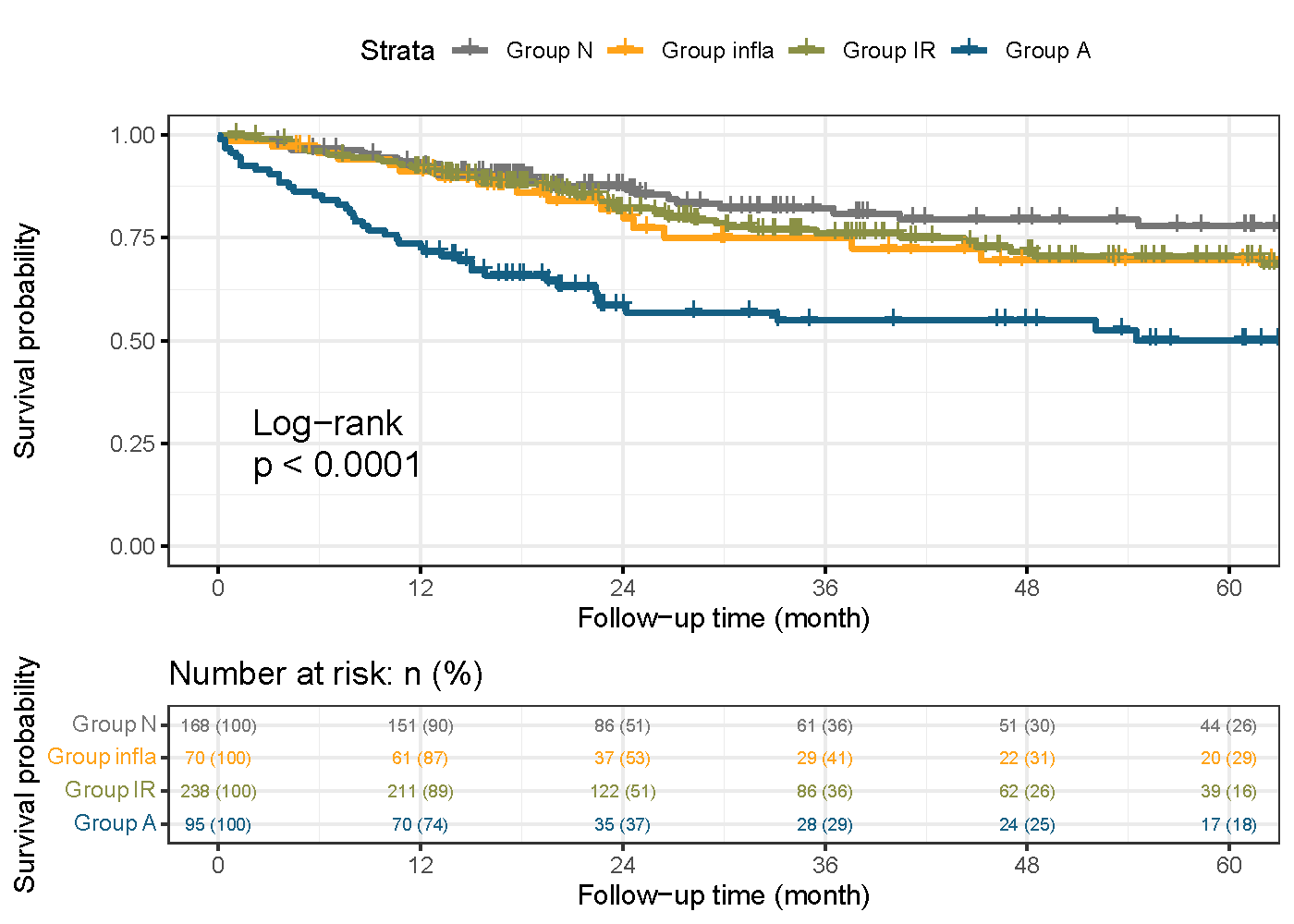


Supplementary Figure 5. The prognostic value of TyG combined with NLR is stronger than the two alone.


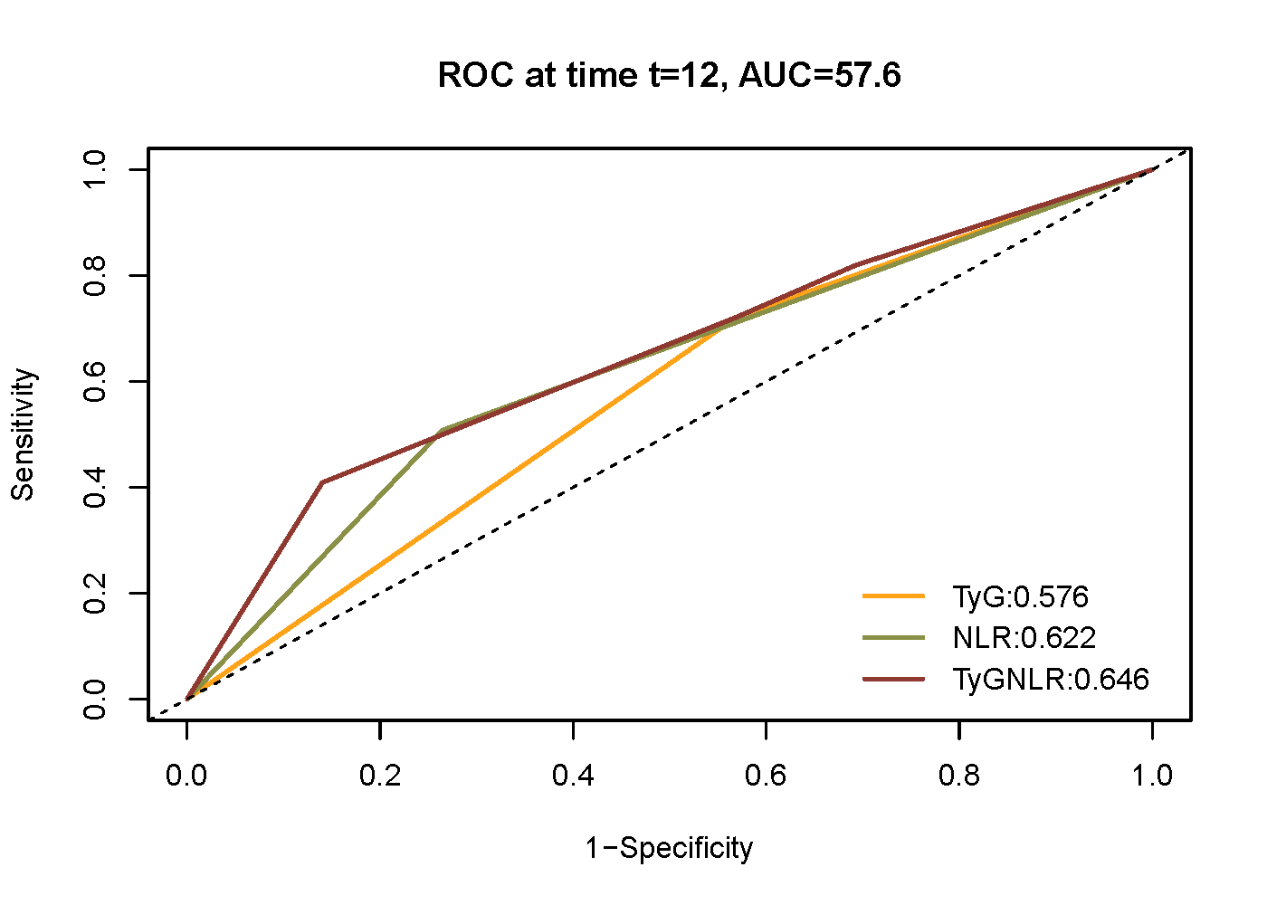

Supplement: Supplementary file 1 — Supporting information. [file IID3-11-e1107-s001.docx]
